# Expression and Purification of Chemokine MIP-3α (CCL20) through a Calmodulin-Fusion Protein System

**DOI:** 10.3390/microorganisms7010008

**Published:** 2019-01-08

**Authors:** Gopal Ramamourthy, Mauricio Arias, Leonard T. Nguyen, Hiroaki Ishida, Hans J. Vogel

**Affiliations:** Biochemistry Research Group, Department of Biological Sciences, University of Calgary, Calgary, AB T2N 1N4, Canada; rgopal@ucalgary.ca (G.R.); ariasm@ucalgary.ca (M.A.); lltnguye@ucalgary.ca (L.T.N.); hishida@ucalgary.ca (H.I.)

**Keywords:** chemokine, MIP-3α, CCL-20, calmodulin-fusion, antimicrobial, antibiofilm

## Abstract

Human macrophage inflammatory protein 3α (MIP-3α), also known as CCL20, is a 70 amino acid chemokine that selectively binds and activates chemokine receptor 6 (CCR6). This chemokine is responsible for inducing the migration of immature dendritic cells, effector, or memory T-cells, and B-cells. Moreover, the MIP-3α protein has been shown to display direct antimicrobial, antiviral and antiprotozoal activities. Because of the potential therapeutic uses of this protein, the efficient production of MIP-3α is of great interest. However, bacterial recombinant production of the MIP-3α protein has been limited by the toxicity of this extremely basic protein (pI 9.7) toward prokaryotic cells, and by solubility problems during expression and purification. In an attempt to overcome these issues, we have investigated the bacterial recombinant expression of MIP-3α by using several common expression and fusion tags, including 6× histidine (His), small ubiquitin modifier protein (SUMO), thioredoxin (TRX), ketosteroid isomerase (KSI), and maltose binding protein (MBP). We have also evaluated a recently introduced calmodulin (CaM)-tag that has been used for the effective expression of many basic antimicrobial peptides (AMPs). Here, we show that the CaM fusion tag system effectively expressed soluble MIP-3α in the cytoplasm of *Escherichia coli* with good yields. Rapid purification was facilitated by the His-tag that was integrated in the CaM-fusion protein system. Multidimensional nuclear magnetic resonance (NMR) studies demonstrated that the recombinant protein was properly folded, with the correct formation of disulfide bonds. In addition, the recombinant MIP-3α had antibacterial activity, and was shown to inhibit the formation of *Pseudomonas aeruginosa* biofilms.

## 1. Introduction

Chemokines are small ~9 kDa signaling proteins that play an important role in chemotaxis, cell-to-cell communication, and the activation of various immune cells [1]. While most chemokines are quite promiscuous and can activate several G protein-coupled chemokine receptors, CCL20, also known as MIP-3α (Macrophage Inflammatory protein 3α), or LARC (Liver Activation Regulated Chemokine), or Exodus-1, is known to bind selectively to the CCR6 (chemokine receptor 6) transmembrane receptor [2,3]. The CCL20/CCR6 interaction is known to play a major role in numerous biological events, such as the activation of dendritic cells [3], lymphocytes [3], and the promotion of intestinal immunity [4], but together, they can also contribute to diseases such as rheumatoid arthritis [5] and psoriasis [6,7], as well as the development of colon cancer [8,9,10,11], and various other cancers [12]. Hence the selective CCL20/CCR6 interaction presents itself as a potential target for pharmaceutical interventions [7].

Apart from their receptor-mediated immune activation properties, several chemokines have been shown to possess direct antibacterial activity [13,14,15], where they act like cationic antimicrobial peptides (AMPs) and perturb bacterial membranes and bind to intracellular targets [16]. Of all the chemokines tested, CCL20 appears to be the most potent in this regard, possibly because of its high lysine and arginine content (pI 9.7) [13]. The amino acid sequence of the 70-residue MIP-3α protein contains 12 positively charged, and four negatively charged residues. Moreover, the electrostatic surface profile of MIP-3α reveals that the positively charged residues are concentrated on the hydrophilic face, forming a basic patch, while most of the solvent-exposed negatively charged moieties are concentrated in another region of MIP-3α [17]. It has been proposed that this positively charged surface region may be important for the anti-infective properties of MIP-3α [17]. Interestingly CCL20 also possesses direct antifungal [18] and antiprotozoal activity [19], as well as potent direct [20] and indirect [21] antiviral activities.

Structure–function relationships for chemokine-receptor interactions are quite well understood [22]. Several nuclear magnetic resonance (NMR) determined solution structures and crystal structures for the murine and human CCL20 homologs have been reported (PDB codes: 5UR7 [7], 1HA6 [23], 2JYO [17], 1M8A [24], 2HCI [25]). While the crystal structure of the CCR6 receptor has yet to be determined, this 7-transmembrane helical protein can be modeled on the basis of the available structures for related G protein-coupled chemokine receptors [26,27]. To facilitate future studies of CCL20 as an activator of CCR6, or as a potential direct antimicrobial agent, a reliable supply of pure protein is required. Here we have evaluated several fusion protein systems in an attempt to biosynthetically produce the correctly folded protein. Our results indicate that the recently introduced calmodulin (CaM)-fusion protein system, which has been successfully used to produce a wide range of cationic AMPs [28], is also suitable for the high-yield expression of correctly folded CCL20/MIP-3α.

## 2. Materials and Methods

### 2.1. Protein Expression and Purification

A synthetic gene for MIP-3α with optimized codons for expression in *Escherichia coli* was purchased from GeneArt (Life Technologies, Carlsbad, CA, USA). The gene for MIP-3α was amplified from the plasmid provided by GeneArt by standard PCR, with primers containing the desired restriction enzyme sites (Supplementary Table S1). The amplified gene was subcloned into several plasmid vectors, as indicated in Supplementary Table S1. The pET15b CaM-TEV (tobacco etch virus) vector was developed to express many peptides and proteins in our laboratory [28], while the other vectors used: (pET 19b containing either 6× histidine (His) or KSI (ketosteroid isomerase), pET SUMO (small ubiquitin modifier protein), pMAL-MBP (maltose binding protein), and pET32a-TRX (thioredoxin)), were commercially obtained. All recombinant plasmids were transformed into the competent *E. coli* strain BL21 (DE3). In addition, the competent *E. coli* Origami B (DE3) strain was transformed with the pET15b-CaM-TEV-MIP-3α construct. We have used this strain because the *E. coli* Origami B (DE3) strain carries mutations in both the thioredoxin reductase (trxB) and glutathione reductase (gor) genes, which delete the activities of trxB and gor, which play important roles for the production of folded proteins containing disulfide bonds in the cytoplasm [29]. The expression, purification, and characterization of CaM-fusion peptides have been discussed in our previous paper [28].

*E. coli* Origami B containing CaM-TEV-MIP-3α was grown in Luria–Bertani (LB) media, in order to purify MIP-3α for antibacterial and antibiofilm assays. For the preparation of the uniformly ^13^C and ^15^N-labeled protein, CaM-TEV-MIP-3α was expressed in minimal M9 medium containing 0.5 g/L ^15^NH_4_Cl, and 3 g/L of ^13^C_6_-glucose. At an optical density (OD) of ~0.6 (measured at 600 nm), the cells were induced with 0.5 mM isopropyl β-D-1-thiogalactopyranoside (IPTG) for 16 h at 28 °C. The cells were collected by centrifugation, and put through at least three passes with a French press (1000 psi) in lysis buffer: 20 mM Tris, 100 mM NaCl, 10 mM imidazole, pH 8. The cell lysate was then clarified by high speed centrifugation (18,000 rpm for 45 min at 6 °C), after which the supernatant was applied onto a column with chelating-Sepharose fast-flow resin (GE Healthcare, Chicago, IL, USA) loaded with NiCl_2_. The column was washed with buffer containing 30 mM imidazole, and the protein of interest was eluted with 400 mM imidazole. The protein-containing fractions were detected by absorbance at 280 nm, and the Bio-Rad protein assay (Bio-Rad Laboratories, Hercules, CA, USA). The fractions were pooled and dialyzed overnight in 4 L of 20 mM Tris/HCl, 100 mM NaCl pH 8.0 at 4 °C. Dialyzed samples were subjected to TEV protease digestion at 34 °C for 16 h in the presence of 0.5 mM ethylenediaminetetraacetic acid (EDTA), a mixture of 30 mM reduced glutathione, and 3 mM oxidized glutathione instead of dithiothreitol (DTT), to preserve the disulfide bonds in MIP-3α. TEV protease was expressed and purified from the pRK793 plasmid (Addgene, Watertown, MA, USA) as previously described [30,31].

Prior to reverse-phase high-performance liquid chromatography (RP-HPLC), the digested mixtures were acidified to pH 3 with trifluoroacetic acid (TFA, usually 0.1%). The MIP-3α protein was purified from a Cosmosil 5C18 AR-300 column (Nacalai Tesque Inc., Kyoto, Japan), running a gradient from buffer A (0.05% TFA in filtered water) to buffer B (0.045% TFA in HPLC-grade acetonitrile). Relevant fractions containing the protein of interest were collected and lyophilized. Protein purity was confirmed by Coomassie Brilliant Blue staining of sodium dodecyl sulfate-polyacrylamide gel electrophoresis (SDS-PAGE). The concentration of MIP-3α was determined by absorbance at 280 nm, using a molar extinction coefficient of 8490 M^−1^·cm^−1^, as determined by ProtParam [32]. The final product contains the cloning artefact Gly-Thr at the *N*-terminal end.

### 2.2. NMR Studies

For NMR experiments ^15^N, or ^13^C, ^15^N-labeled CCL20 protein was prepared by the expression of the fusion protein in M9 minimal containing 0.5 g ^15^NH_4_Cl per liter and 3 g ^13^C_6_-glucose. The NMR samples contained 0.5 mM protein dissolved in 90% H_2_O/10% D_2_O at pH 4.2 [23]. All NMR experiments were performed at 25 °C on a Bruker Avance 700 MHz NMR spectrometer equipped with a triple resonance TXI probe with a single axis z-gradient. Backbone assignments were obtained by collecting 3D HNCACB, CBCA(CO)NH, HNCO, HN(CA)CO, HNCA, and HN(CO)CA experiments, as well as 2D, ^1^H, ^15^N heteronuclear single quantum coherence (HSQC) spectra. Heteronucler {^1^H}-^15^N nuclear Overhauser effect (NOE) dynamics data were obtained as well, to study the protein dynamics. The chemical shift index was calculated according to a published methods [33]. All NMR spectra of MIP-3α were processed using NMRPipe [34] and analyzed with NMRView [35].

### 2.3. Minimum Inhibitory Concentration (MIC) and Minimum Biofilm Inhibitory Concentration (MBIC)

Crystal violet staining of adherent biofilms was used to determine the MBIC values for MIP-3α and tobramycin, using a broth microdilution method with minor modifications [36]. Two-fold serial dilutions of test agents were prepared in 100 μL of 10% LB solution in the wells of a 96-well flat-bottom microtiter plate (Sigma-Aldrich, St Louis, MO, USA), followed by the addition of 100 μL of *Pseudomonas aeruginosa* bacterial suspension (i.e., 1.0 × 10^7^ CFU/mL) [37]. This resulted in a final inoculum of 5 × 10^6^ CFU/mL in each well, and the final concentrations of the agent ranged from 0.25 to 128 µM. After incubation of the plates for 24 h at 37 °C, the MICs were determined for MIP-3α and tobramycin by measuring growth inhibition at 620 nm using an Eppendorf PlateReader AF2200 (Eppendorf, Mississauga, ON, Canada) [37]. After the MIC determinations, the planktonic cells (culture supernatant) were discarded, and the extent of biofilm formation was determined by the use of the crystal violet stain [36]. In brief, the wells were carefully rinsed with sterile saline (0.9% NaCl *w*/*v*) to eliminate free-floating bacteria. The biofilm that was attached to the wells in the plate was stained with 0.1% (*w*/*v*) crystal violet solution for 30 min, after which the excess stain was thoroughly rinsed away with distilled water until the negative-control wells appeared colorless. Finally, 200 μL of 95% ethanol was added to each well, and the OD_600_ of the stained biofilm was measured with an Eppendorf PlateReader AF2200 (Eppendorf, Mississauga, ON). The MBIC values for peptides were measured based on the lowest concentration that showed 100% inhibition of biofilm-forming bacteria adhering to the surface [36].

### 2.4. Minimum Biofilm Reduction Concentration (MBRC)

Exactly 150 μL of inoculum cells (i.e., 5 × 10^6^ CFU/mL) was transferred into each well of a 96-well microtiter plate (Sigma-Aldrich, St Louis, MO, USA) for biofilm cultivation [38]. The plate was incubated overnight in a humidifier chamber at 37 °C to allow biofilm formation on the surfaces of 88 wells. As a control, pure medium was added to some wells in the first column of a 96-well plate [38]. Using a multichannel pipette (20–200 μL), the planktonic cells were discarded without disturbing the adherent biofilm cells on the surface, and the bottom surface of each well was washed with saline prior to susceptibility testing. A MIP-3α stock solution was diluted stepwise (concentrations: 128, 64, 32, 16, 8, 4, 2, 1, 0.5, and 0.25 µM) in 10% LB solution. The serially diluted protein solutions in 10% LB were added to the biofilm suspension in the 96-well plate, and then incubated for a second night at 37 °C and 80% relative humidity [38]. The wells of the plate were washed free of media and planktonic bacteria cells with saline, before stepwise crystal violet staining, as described in the MBIC section [36]. The MBRC was defined as the minimum concentration of compound that reduced 100% of the biofilm formation [36].

### 2.5. Minimum Biofilm Eradication Concentration (MBEC)

For the determination of the MBEC value for tobramycin and MIP-3α, biofilms were cultivated in a Calgary Biofilm device as described previously [38,39]. All 96 wells of the device were seeded with *P. aeruginosa* strains at a concentration of 1 × 10^6^ CFU/mL in 10% LB, after which biofilms were allowed to form for 24 h at 37 °C with shaking. To quantify the biofilms formed, the pegs of the device were removed from the wells following the 24 h incubation, and any free-floating planktonic cells were removed by washing the pegs of the device with phosphate-buffered saline (PBS). Mean viable cell counts were determined for four pegs according to the established methods [38]. To examine the eradication effect of MIP-3α and tobramycin on biofilm growth, MIP-3α (128–0.25 µM) and tobramycin (128–0.25 µM) were made up in 10% LB in 96-well microtiter plates, according to standard protocols [38]. Biofilms on the pegs of the device were inserted into these plates. After a 24 h exposure at 37 °C under static conditions, the biofilms were rinsed, and cells were plated for viable cell counting, as previously described [38]. The MBEC was defined as the minimum concentration of compound that eradicated 100% of the biofilm cells.

## 3. Results and Discussion

### 3.1. Expression of MIP-3α from Different Constructs

**Figure 1:**
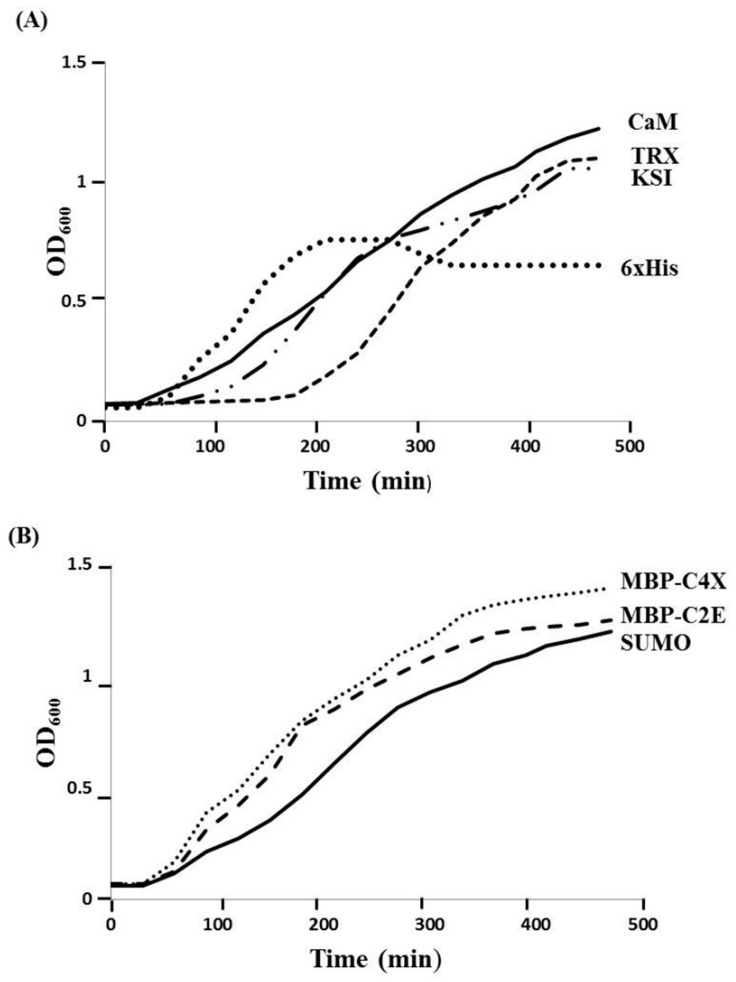
The effect of IPTG-induced MIP-3α expression with various fusion systems on the growth of the *E. coli* BL21 (DE3), and Origami (DE3) host cells. (**A**) The growth curves obtained with pET15b-CaM-MIP-3α (solid line), pET19b-6× His-MIP-3α (round dot), and pET32a-TRX-MIP-3α (dashed line), pET19b-KSI-MIP-3α (long dash-dot-dot), and (**B**) The growth curves obtained with pET-SUMO-MIP-3α (solid line), pMAL-C4X-MIP-3α (round dot), pMAL-C2E-MIP-3α (dash line) are also shown for comparison. *E. coli* cells were grown in 5 mL of LB medium at 37 °C and induced with 0.5 mM IPTG at an OD_600_ ~ 0.65.

**Figure 2:**
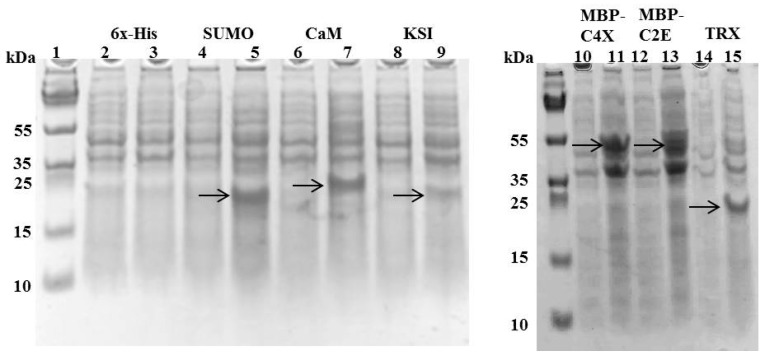
SDS-PAGE showing the expression of different tags with MIP-3α constructs after 4 h of IPTG induction at 37 °C. Even-numbered lanes show the cultures before IPTG induction, odd-numbered lanes show the induced constructs of pET19b-6× His-MIP-3α (Lanes 2–3), pET-SUM0-MIP-3α (Lanes 4–5), pET15b-CaM-MIP-3α (Lanes 6–7), pET19b-KSI-MIP-3α (Lanes 8–9), pMAL-C4X-MIP-3α (Lanes 10–11), pMAL-C2E-MIP-3α (Lanes 12–13), and pET32a-TRX-MIP-3α (Lanes 14–15). The bands observed for MIP-3α attached to different fusion proteins are indicated by the arrows.

Different MIP-3α expression constructs were designed, in order to evaluate the effects of distinct fusion tags on the expression of the MIP-3α protein, as well as the viability of the *E. coli* host. In our initial experiments, we monitored the growth of the *E. coli* cultures that expressed MIP-3α with different tags. Figure 1 shows the growth curves in LB media for *E. coli* transformed with different plasmid constructs (Supplementary Table S1). After induction by IPTG, the OD_600_ for the *E. coli* cells was measured for at least 4 h. The IPTG-induced expression of MIP-3α fused to the CaM-tag was compared for the 6× His, KSI, MBP, SUMO, and TRX-fusion expression systems (Figure 1). The results showed that almost all of the expression systems allowed the cells to grow during the mid-logarithmic phase after IPTG induction. One notable exception was the 6× His-MIP-3α (pET19b-MIP-3α) construct, which inhibited cell growth after 1.5 h of induction, reaching only OD_600_ ~ 0.75. However, unlike what was seen for the expression of AMP [28], all of the other *E. coli* cells expressing MIP-3α fusion proteins displayed an uninterrupted exponential growth phase reaching OD_600_ > 1.2 (Figure 1). Overexpression of MIP-3α by all constructs was confirmed by SDS-PAGE (Figure 2). There was no detectable expression of MIP-3α in the pET19b–MIP-3α system, as seen by SDS-PAGE (Figure 2). These results indicate that 6× His-MIP-3α (pET19b-MIP-3α) is toxic to the host cells, while all other fusion tags tested were tolerated by the cells.

### 3.2. Expression Levels and Purification of Different Tags-Fused MIP-3α in E. coli

**Figure 3:**
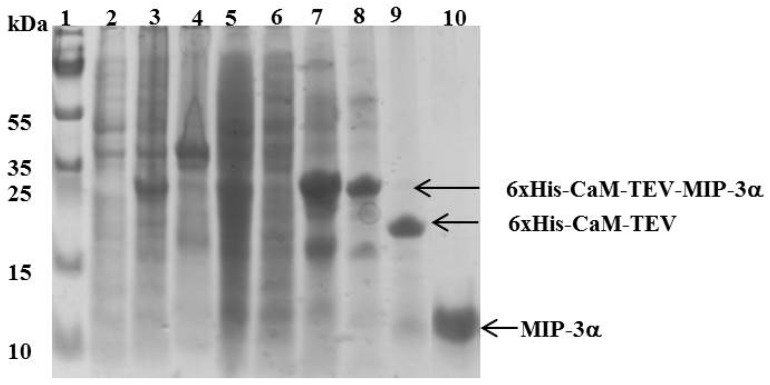
SDS-PAGE showing the expression and purification of MIP-3α. Lane 1 shows the molecular mass marker. Lanes 2 and 3 represent the *E. coli* cell lysate before and after IPTG induction, respectively. Lanes 4 and 5 represent the pellet and the supernatant of the cell lysate, respectively after a high speed centrifugation (18,000 rpm for 45 min at 6 °C). Lane 6 represents the unbound protein eluted from the Ni^2+^-column. Lane 7 represents the peak fraction eluted from the Ni^2+^-column with 400 mM imidazole. In lane 8, the elution fraction was dialyzed against 20 mM Tris HCl pH 7.8 and 100 mM NaCl. Lane 9 shows the dialyzed sample digested with TEV protease. Lane 10 shows the final MIP-3α purified by HPLC.

In addition to promoting the survival of *E. coli* cells when expressing MIP-3α, the different fusion tags used in this study also influenced the solubility and expression levels of MIP-3α following IPTG induction. The cell lysates of the *E. coli* cells obtained after induction and cell lysis indicated that the CaM-fused MIP-3α protein was present in the supernatant (Figure 3). In all other cases, the fused MIP-3α was mainly found in the pellet (as inclusion bodies) after high-speed centrifugation (Supplementary Figures S1–S3). The SUMO-tag system has previously been proposed for the effective expression of many AMPs [40]. However, in our case, the SUMO-fusion system gave rise to expression of MIP-3α into inclusion bodies (Supplementary Figure S1). Similarly, KSI was used here as a fusion tag [41], with the knowledge that it will induce the targeting of MIP-3α to inclusion bodies (Supplementary Figure S3). The purification of MIP-3α from the inclusion bodies should be possible, but in our experience, it is a highly time consuming and often inefficient process, which involves affinity chromatography after protein denaturation with chaotropic agents (urea and guanidium-HCl), followed by protein refolding [42,43,44]. Nonetheless, in the case of MBP-MIP-3α, a small amount of soluble fusion protein could be recovered (Supplementary Figure S4). Purification of the soluble MBP-MIP-3α fusion protein was achieved on an amylose resin, but a very low yield was obtained. Additionally, we noted that the release of MIP-3α from the MBP by Factor Xa digestion produced an insoluble form of MIP-3α (Supplementary Figure S4).

**Figure 4:**
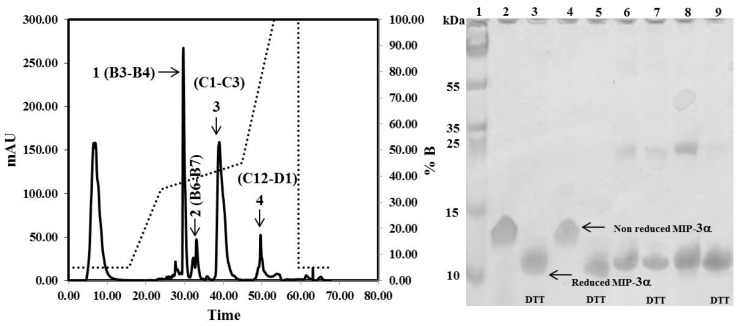
RP-HPLC chromatograms obtained for the purification of MIP-3α after TEV protease digestion. Indicated by the arrows are the purified MIP-3α (1), MIP-3α and cleaved CaM (2), cleaved CaM (3), and the remaining undigested CaM-MIP-3α construct (4). Solid lines represent the absorbance at 280 nm. The dotted line represents the acetonitrile (buffer B) gradient. SDS-PAGE showing the purification of MIP-3α. Lanes 2, 4, 6, and 8 shows fraction B3, B4, B6, and B7 respectively. Lanes 3, 5, 7, and 9 shows fraction B3, B4, B6, and B7 respectively, were incubated with 10 mM DTT at 37 °C for 1 h before SDS-PAGE analysis.

The CaM-TEV-MIP-3α construct contains an *N*-terminal 6× His-tag [28], and as expected, purification of the fusion protein could be achieved directly by standard nickel-nitrilotriacetic acid (Ni-NTA)-column chromatography. The amount of CaM-MIP-3α protein that was eluted from this step was 45–60 mg per liter of culture. After an overnight dialysis step into TEV digestion buffer, the purified protein was subjected to TEV protease cleavage. After digestion, the isolated protein could be separated from CaM and TEV protease by RP chromatography, using a C18 HPLC column (Figure 4). The HPLC fractions were separated and lyophilized for purity confirmation. The SDS-PAGE gels highlighted that the fractions of B3 and B4 corresponded to the MIP-3α band, while the fractions B6 and B7 contained a mixture of MIP-3α and CaM (Figure 4). Additionally, fractions B3 and B4 were eluted at approximately 30 min using a linear gradient of 5–60% acetonitrile in aqueous solution containing 0.05% (*v*/*v*) TFA in RP-HPLC chromatogram (Figure 4), while CaM binds to the column under these conditions. In this case, the RP-HPLC pattern for the purification of MIP-3α from CaM and TEV protease indicated that the less hydrophobic MIP-3α was eluted before CaM.

It is important to note here that the molecular weight of the recombinant MIP-3α protein, containing 70 amino acids, is approximately 8.0 kDa. However, the SDS-PAGE migration pattern of the MIP-3α-containing fractions showed that the DTT-reduced protein settled at approximately 11 kDa, and the non-reduced protein, at approximately 13 kDa (Figure 4).

In addition, the *E. coli* strain BL21 also expresses the CaM-TEV-MIP-3α in the soluble fraction (Supplementary Figure S5), but a lot of MIP-3α exists in the form of mis-folded proteins that form aggregates after the cleavage of the tag. However, a small amount of soluble fraction of MIP-3α was acquired after the cleavage of the tag, and also showed a proper folding pattern, as determined by NMR (data not shown, see below). Altogether, the expression of CaM-TEV-MIP-3α in both *E. coli* BL21 and *E. coli* Origami cells were successful, but the use of the *E. coli* Origami cells increased the total amount of correctly folded MIP-3α in the soluble fraction when compared to the *E. coli* BL21. The lack of a reducing environment [29] in the Origami cells is likely responsible for this observation.

### 3.3. NMR Experiments

**Figure 5:**
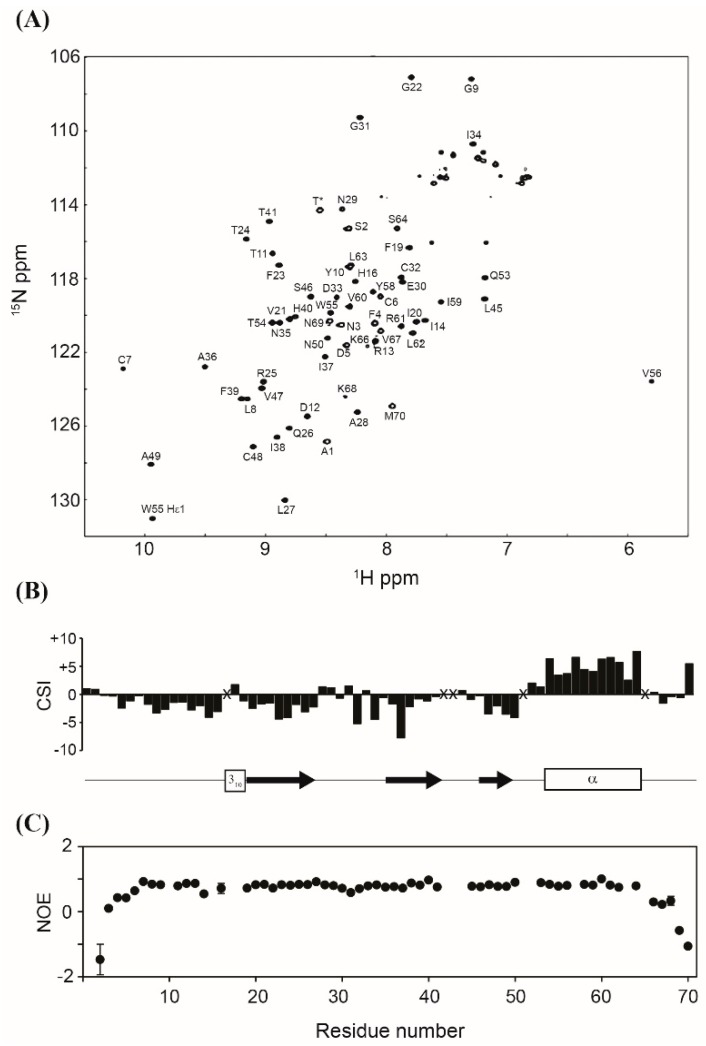
(**A**) Assigned ^1^H, ^15^N-HSQC NMR spectrum of MIP-3α. The signal marked with T* originates from the extra Thr residue in the cloning artefact. (**B**) Secondary shifts were calculated as the added differences between the observed and the random coil chemical shifts of the Cα and C′ atoms, and they are plotted as a function of the residue number. The positions of the secondary structure in the previously reported structure of MIP-3α (PDB code: 2jyo) are also shown. (**C**) ^1^H, ^15^N-heteronuclear NOE values are plotted as a function of residue number. X indicates that assignments were not available.

Obtaining a properly folded protein is crucial for successful recombinant protein expression. Therefore, the structure of the purified MIP-3α was assessed by multinuclear multidimensional NMR spectroscopy. Figure 5A shows the assigned ^1^H, ^15^N-HSQC NMR spectrum of the purified ^13^C, ^15^N-labeled MIP-3α. Although the amide signals of L15, K18, K42, K43, K44, K52, K57, and K65 were not observed under our experimental conditions, all other backbone amide signals except for two prolines were unambiguously assigned in the HSQC spectrum. The NH chemical shifts were consistent with those previously reported [23]. The Cβ chemical shifts of all four Cys residues, including C6, C7, C32, and C48, appeared in their oxidized positions (~40 ppm), indicating that all Cys residues are involved in the formation of disulphide bonds. The secondary structures predicted from the chemical shifts of the Cα and C′ atoms are identical with those reported for the solution structure of MIP-3α (PDB code: 2jyo; [23]) (Figure 5B). The {^1^H}-^15^N hetero-nuclear NOE data showed that MIP-3α does not contain any flexible region throughout the structure, except for both termini (Figure 5C), which is also in excellent agreement with the reported structure. These results confirm that our recombinant MIP-3α protein, purified using the CaM-fusion system, is properly folded with the two intramolecular disulphide bonds. Previously, we have shown that the CaM-fusion system also successfully promoted the folding of the HBD-3 human defensin protein, which contains three intramolecular disulphide bonds [28].

When the CaM-fusion system was used to successfully express several cationic AMPs, it was demonstrated that all AMPs could bind to CaM, which likely gave rise to the reduced toxicity of the expressed AMPs against the bacterial host cells, and afforded the protection of the AMPs from degradation during expression and purification [28]. In this study, we therefore investigated whether the MIP-3α protein could bind to CaM, by recording ^1^H, ^15^N-HSQC NMR spectra of ^15^N-labeled MIP-3α, and titrating with unlabeled CaM (data not shown). Titrations were carried out in the presence and absence of calcium. In both titrations, chemical shift changes were observed in the NMR spectra, indicating that calcium-free CaM as well as calcium–CaM can bind to MIP-3α. Extensive line broadening was observed in the course of these NMR titrations, which was indicative of intermediate exchange on the NMR timescale. Such behavior is normally observed for Kds in the low micromolar range. Moreover, these data suggest that the interactions between CaM and MIP-3α, may have masked the antimicrobial properties of MIP-3α, like the other AMPs [28].

### 3.4. Effects of MIP-3α on Planktonic Cells and Biofilms of Pseudomonas aeruginosa

**Figure 6:**
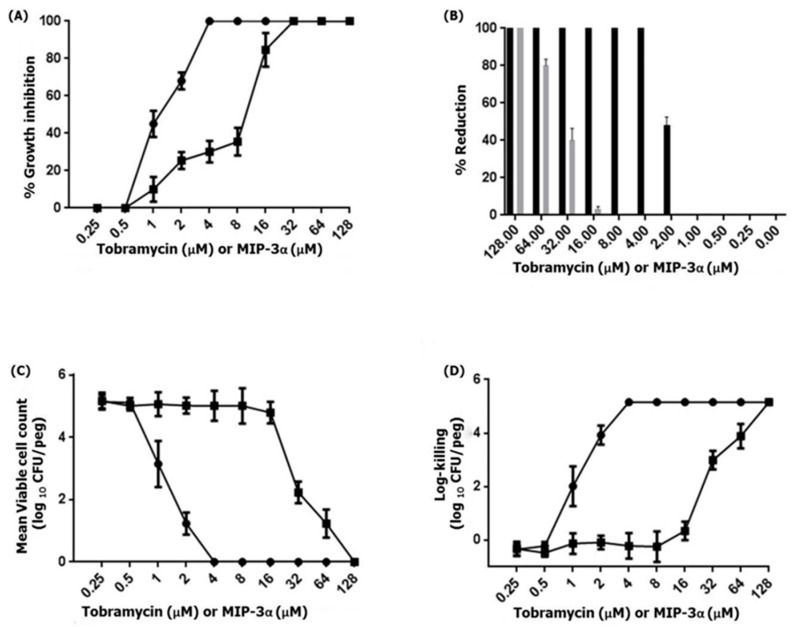
(**A**) The MBIC of tobramycin (circles) or MIP-3α (squares) was determined by the standard crystal violet assay for the quantification of growth inhibition. (**B**) The MBRC of tobramycin (black) or MIP-3α (gray) was determined by an assay with crystal violet, and percentage reduction of the 24-h-old-*P. aeruginosa* PAO1 biofilm resulting from MIP-3α or tobramycin at 10 dosage levels with 24 h treatment exposures. (**C**,**D**) The MBEC of tobramycin (circles) or MIP-3α (squares) was determined by an assay with the Calgary biofilm device. *P. aeruginosa* PAO1 biofilms were exposed to tobramycin or MIP-3α for 24 h. The killing of *P. aeruginosa* PAO1 biofilms were determined by plate counts.

Previous studies have demonstrated that MIP-3α exhibits antimicrobial activity against various Gram-negative and Gram-positive bacterial as well as fungal strains [13,14,15,17,18]. Therefore, we examined the antipseudomonal activity of MIP-3α against the biofilm-forming *P. aeruginosa* PAO1 strain. This results indicated that MIP-3α and tobramycin showed antipseudomonal activity against *P. aeruginosa*, at a concentration of 16 µM and 2 µM, respectively. Importantly, biofilms produced by *P. aeruginosa* frequently cause life-threatening infections in individuals with compromised immune systems or cystic fibrosis [45]. Therefore, we further examined the ability of MIP-3α to interfere in biofilm formation. The MBIC_100_ was determined based on the percent reduction of crystal blue when compared to an untreated control. Figure 6 shows the MBIC_100_ values for MIP-3α and tobramycin obtained after 24 h of treatment. *P. aeruginosa* PAO1 was found to be susceptible to tobramycin, as indicated by MBIC_100_ values in the range of 2–4 µM, while MIP-3α showed some potency against *P. aeruginosa*, with MBIC_100_ values being estimated to be between 16 and 32 µM. Next, we performed MBRC experiments to analyze the biofilm reduction effects of the control tobramycin and MIP-3α on the *P. aeruginosa* biofilm. Figure 6B shows the percentage reduction of biofilm after treatment with tobramycin, or MIP-3α. Tobramycin treatments provided a 100% reduction in biofilm formation at a concentration of 4 µM, while MIP-3α treatments could reduce biofilm formation by 75% to 80% at a concentration of 64 µM. Finally, the MBECs of tobramycin and MIP-3α were determined at concentrations of 4 and 128 µM, respectively. Several encouraging findings have shown that various AMPs, including LL-37, MUC7, G10KHc, colistin, K_4_-S4(1-15)a, and dhvar4a, showed antibiofilm and eradication activity against *P. aeruginosa*, *S. mutans*, and several other pathogenic bacteria [36,46,47,48,49]. In this context, the eradication effect exerted by MIP-3α against drug-resistant strains of *P. aeruginosa* would be of particular significance, as it may be useful for patients suffering from cystic fibrosis and implant infections.

## 4. Conclusions

In conclusion, several expression systems were designed for the production of MIP-3α with different fusion proteins or tags. We found that expression of pET19b-MIP-3α without any partner protein is toxic to *E. coli*. Other protein fusion tags also had disadvantages, as inclusion bodies or insoluble forms of MIP-3α were typically obtained during expression or protein purification. Fortunately, expression of MBP-MIP-3α and CaM-fusion-MIP-3α generated a soluble form of MIP-3α, but the smaller acidic CaM-fusion protein (16.7 kDa; pI 4.1) provided a very good yield compared to the larger and basic MBP (31.8 kDa; pI 7.9). We were successful in obtaining fully isotope-labeled MIP-3α, and we demonstrated that the protein was correctly folded, by NMR spectroscopy. Moreover, we could demonstrate by NMR spectroscopy that MIP-3α and CaM can bind to each other, and that this may have contributed to the enhanced stability during the expression and purification. Overexpression and purification of MIP-3α through the CaM-fusion system also gave rise to a MIP-3α protein that displayed antimicrobial activity, and possessed biofilm inhibition and biofilm eradication activity against *P. aeruginosa*. Importantly, the expression levels of MIP-3α with the CaM-fusion tag could be further optimized, which would make it useful for many applications, including potentially the production of MIP-3α for providing antimicrobial and antibiofilm activity at wound sites.

## Supplementary Materials

The following are available online at http://www.mdpi.com/2076-2607/7/1/8/s1, Figure S1: Solubility of SUMO-MIP-3α expressed in *E. coli* by BL21(pET-SUMO-MIP-3α) after cell lysis (French Press 1000 PSI × 3) and high-speed centrifugation (18,000 rpm for 45 min at 6 °C) followed by the purification of soluble SUMO-MIP-3α by nickel affinity chromatography. These results indicate that most of the SUMO-MIP-3α fusion protein is expressed in inclusion bodies, and is present in the pellet after cell lysis, Figure S2: Solubility of TRX-MIP-3α expressed in *E. coli* BL21(pET32a-MIP-3α) after cell lysis (French Press 1000 PSI × 3) and high-speed centrifugation (18,000 rpm for 45 min at 6 °C), followed by the purification of soluble TRX-MIP-3α by nickel affinity chromatography. These results indicate that most of the TRX-MIP-3α fusion protein is expressed as inclusion bodies and only a small fraction is produced as soluble protein, Figure S3: Solubility of KSI-MIP-3α expressed in *E. coli* BL21 (pET19b-KSI-MIP3α) after cell lysis (French Press 1000 PSI × 3) and high-speed centrifugation (18,000 rpm for 45 min at 6 °C). These results indicate that as expected [50], most of the KSI-MIP-3α fusion protein is expressed as inclusion bodies, and is present in the pellet after cell lysis, Figure S4: Solubility of MBP-MIP-3α expressed by BL21(pMAL-C4X-MIP-3α) after cell lysis (French Press 1000 PSI × 3) and high-speed centrifugation (18,000 rpm for 45 min at 6 °C) followed by the purification of soluble MBP-MIP-3α by amylose affinity chromatography (left). These results indicate that the MBP-MIP-3α fusion protein is expressed as inclusion bodies as well as soluble protein. However, in addition to the fusion protein, the MBP alone is also expressed. Digestion of the MBP-MIP-3α fusion protein with Factor Xa (right). These results showed that after fusion protein digestion, the MIP-3α became insoluble and was observed in the pellet of the digestion reaction, likely reflecting the production of a protein that was not correctly folded, Figure S5: SDS-PAGE showing the expression and purification of MIP-3α in *E. coli* BL21. Lane 1 shows the molecular mass markers. Lanes 2 and 3 represent the *E. coli* BL21 cell lysate before and after IPTG induction, respectively. Lanes 4 and 5 represent the pellet and the supernatant of the cell lysate (French Press 1000 PSI × 3), respectively, after high-speed centrifugation (18,000 rpm for 45 min at 6 °C). Lane 6 represents the peak fraction eluted from the Ni^2+^-column with 400 mM imidazole. Lane 7 shows proteins after the dialyzed sample was digested with TEV protease. Lanes 8 and 9 shows the final MIP-3α (B3 and B4) with and without reduced agent, respectively. Lane 10 and 11 shows proteins from the pooled fractions (B6 and B7) with and without reduced agents, respectively. Lane 12 shows proteins from the pooled fractions (C1, C2 and C3). Lane 13 shows proteins from the pooled fractions (C11, C12 and D1). The RP-HPLC chromatogram obtained from expression in *E. coli* BL21 (data not shown), was similar to that obtained with the *E. coli* Origami strain (Figure 4), Table S1: *E. coli* transformed with different plasmid constructs.

## Abbreviations

MIP-3α	macrophage inflammatory protein-3α
LARC	liver and activation-regulated chemokine
CCR6	chemokine receptor 6
AMPs	antimicrobial peptides
NMR	nuclear magnetic resonance
CaM	calmodulin
TEV	tobacco etch virus
KSI	ketosteroid isomerase
SUMO	small ubiquitous modifier protein
MBP	maltose binding protein
TRX	thioredoxin
TrxB	thioredoxin reductase
Gor	glutathione reductase
LB	Luria–Bertani
OD	optical density
IPTG	isopropyl β-ᴅ-thiogalactopyranoside
EDTA	ethylenediaminetetraacetic acid
DTT	dithiothreitol
RP-HPLC	reverse phase-HPLC, high-performance liquid chromatography
TFA	trifluoroacetic acid
SDS-PAGE	sodium dodecyl sulphate-polyacrylamide gel electrophoresis
HSQC	heteronuclear single quantum coherence
NOE	nuclear Overhauser effect
MIC	minimum inhibitory concentration
MBIC	minimum biofilm inhibitory concentration
MBRC	minimum biofilm reduction concentration
MBEC	minimum biofilm eradication concentration
PBS	phosphate-buffered saline
Ni-NTA	nickel-nitrilotriacetic acid
