# Supplementary material for: Expression and Purification of Chemokine MIP-3α (CCL20) through a Calmodulin-Fusion Protein System
Source: Microorganisms. 2019 Jan 8;7(1):8. doi: 10.3390/microorganisms7010008 (PMC6352211; doi:10.3390/microorganisms7010008)
Supplement: Supplementary file 1 [file microorganisms-07-00008-s001.pdf]

microorganisms

Supplementary Materials

Table S1. E. coli transformed with different plasmid constructs.

Plasmids	*Restrictions sites*	*Tag/Expression/Protease*
pET15b-CaM-TEV-MIP-3α	*KpnI/Xho I*	*6xHis-CaM/Cytoplasm/TEV*
pET19b-MIP-3α	*NdeI/ Xho I*	*6xHis (N-terminal)/Cytoplasm/Enterokinase*
pET19b-KSI-MIP-3α	*NdeI/ Xho I*	*6xHis-KSI/Cytoplasm/Enterokinase*
pET-SUMO-MIP-3α	*NdeI/ Xho I*	*6xHis-SUMO/Cytoplasm/SUMO Protease*
pMAL-C2E-MIP-3α	*KpnI/Hind III*	*MBP/Cytoplasm/Enterokinase*
*pMAL-C4X-MIP-3α**	*XmnI/HindIII*	*MBP/Cytoplasm/Endoprotease*
pET32a-TRX-MIP-3α	*NcoI/XhoI*	*Thioredoxin/Cytoplasm/Enterokinase*

* Mutation (A312V) in MBP that showed improved affinity and solubility properties [1].

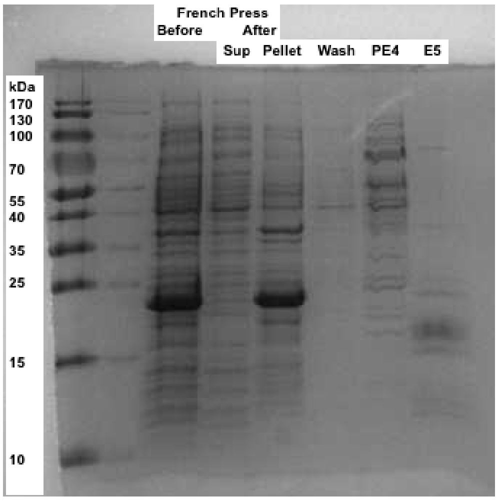

Figure S1. *Solubility of SUMO-MIP-3α* expressed in E. coli by BL21(pET-SUMO-MIP-3α) after cell lysis (French Press 1000PSI x 3) and high speed centrifugation (18000 rpm for 45 min at 6°C) followed by purification of soluble SUMO-MIP-3α by nickel affinity chromatography. These results indicate that most of the SUMO-MIP-3α fusion protein is expressed in inclusion bodies and is present in the pellet after cell lysis.

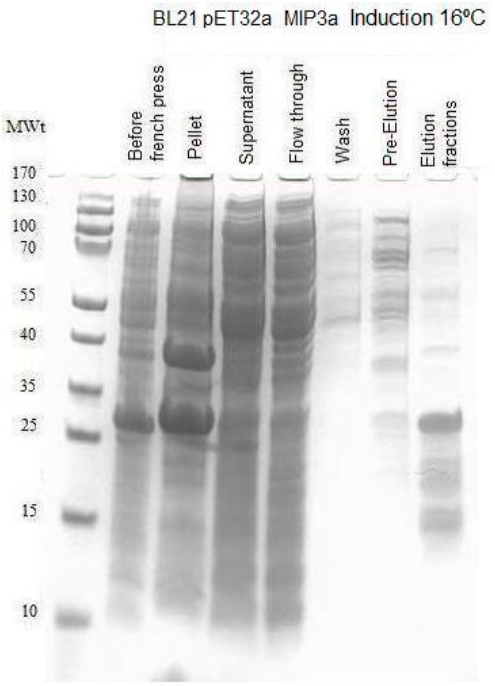

Figure S2. Solubility of TRX-MIP-3α expressed in E. coli BL21(pET32a-MIP-3α) after cell lysis (French Press 1000PSI x 3) and high speed centrifugation (18000 rpm for 45min at 6°C) followed by purification of soluble TRX-MIP-3α by nickel affinity chromatography. These results indicate that most of the TRX-MIP-3α fusion protein is expressed as inclusion bodies and only a small fraction is produced as soluble protein.

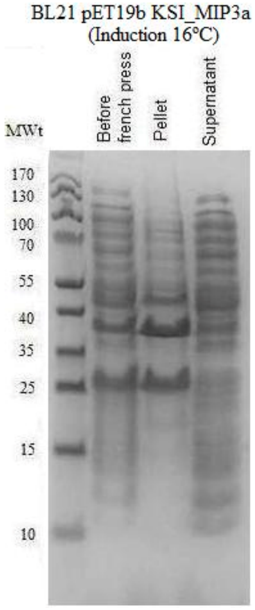

Figure S3. *Solubility of KSI-MIP-3α expressed in E. coli BL21 (pET19b-KSI-MIP3α) after cell lysis (French Press 1000PSI x 3) and high speed centrifugation (18000 rpm for 45min at 6°C). These results indicate that as expected [2] most of the KSI-MIP-3α fusion protein is expressed as inclusion bodies and is present in the pellet after cell lysis.*

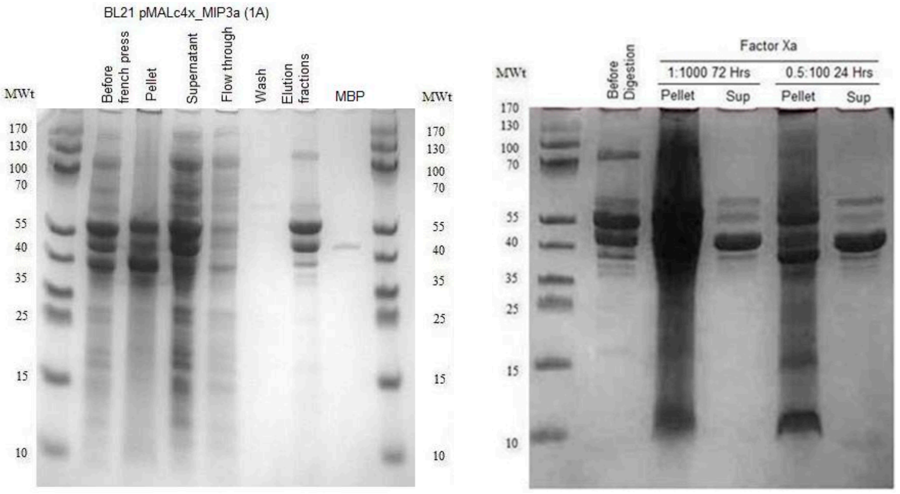

Figure S4. Solubility of MBP-MIP-3α expressed by BL21(pMAL-C4X-MIP-3α) after cell lysis (French Press 1000PSI x 3) and high speed centrifugation (18000 rpm for 45min at 6°C) followed by purification of soluble MBP-MIP-3α by amylose affinity chromatography (left). These results indicate that MBP-MIP-3α fusion protein is expressed as inclusion bodies as well as soluble protein. However, in addition to the fusion protein, the MBP alone is also expressed. Digestion of the MBP-MIP-3α fusion protein with Factor Xa (right). These results showed that after fusion protein digestion, the MIP-3α became insoluble and was observed in the pellet of the digestion reaction, likely reflecting the production of a protein that was not correctly folded.

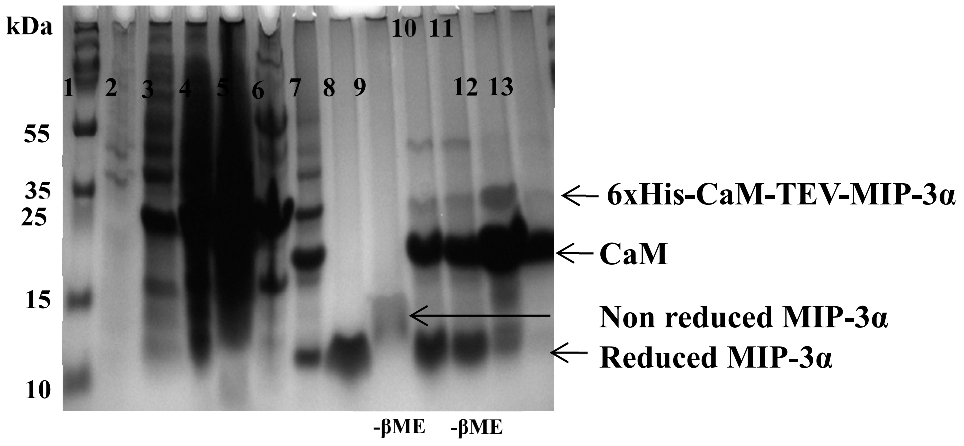

Figure S5. SDS-PAGE showing the expression and purification of MIP-3α *in* E. coli BL21. Lane 1 shows the molecular mass markers. Lanes 2 and 3 represent the E. coli BL21 cell lysate before and after IPTG induction, respectively. Lane 4 and 5 represents the pellet and the supernatant of the cell lysate *(French Press 1000PSI x 3), respectively* after *high speed centrifugation (18000 rpm for 45min at 6°C).* Lane 6 represents the peak fraction eluted from the Ni^{2+}-column with 400 mM imidazole. Lane 7 shows proteins after the dialyzed sample was digested with TEV protease. Lanes 8 and 9 shows the final MIP-3α (B3 and B4) with and without reduced agent, respectively. Lane 10 and 11 shows proteins from the pooled fractions (B6 and B7) with and without reduced agents, respectively. Lane 12 shows proteins from the pooled fractions (C1, C2 and C3). Lane 13 shows proteins from the pooled fractions (C11, C12 and D1). The RP-HPLC Chromatogram obtained from expressed in E. coli BL21 (data not shown), was similar to that obtained with the E. coli Origami strain (Figure 4).

References

1. Walker, I.H.; Hsieh, P.C.; Riggs, P.D. Mutations in maltose-binding protein that alter affinity and solubility properties. *Appl. Microbiol. Biotechnol.* **2010**, 88, 187–197.
2. Zorko, M.; Japelj, B.; Hafner-Bratkovic, I.; Jerala, R. Expression, purification and structural studies of a short antimicrobial peptide. *Biochem. Biophys. Acta* **2009**, *1788*, 314–323.
